# Pulmonary Delivery of Biological Drugs

**DOI:** 10.3390/pharmaceutics12111025

**Published:** 2020-10-26

**Authors:** Wanling Liang, Harry W. Pan, Driton Vllasaliu, Jenny K. W. Lam

**Affiliations:** 1 Department of Pharmacology and Pharmacy, Li Ka Shing Faculty of Medicine, The University of Hong Kong, 21 Sassoon Road, Pokfulam, Hong Kong, China; hwpan@connect.hku.hk (H.W.P.); jkwlam@hku.hk (J.K.W.L.); 2 School of Cancer and Pharmaceutical Sciences, King’s College London, 150 Stamford Street, London SE1 9NH, UK; driton.vllasaliu@kcl.ac.uk

**Keywords:** aerosol, inhalation, lung, monoclonal antibodies, therapeutic proteins

## Abstract

In the last decade, biological drugs have rapidly proliferated and have now become an important therapeutic modality. This is because of their high potency, high specificity and desirable safety profile. The majority of biological drugs are peptide- and protein-based therapeutics with poor oral bioavailability. They are normally administered by parenteral injection (with a very few exceptions). Pulmonary delivery is an attractive non-invasive alternative route of administration for local and systemic delivery of biologics with immense potential to treat various diseases, including diabetes, cystic fibrosis, respiratory viral infection and asthma, etc. The massive surface area and extensive vascularisation in the lungs enable rapid absorption and fast onset of action. Despite the benefits of pulmonary delivery, development of inhalable biological drug is a challenging task. There are various anatomical, physiological and immunological barriers that affect the therapeutic efficacy of inhaled formulations. This review assesses the characteristics of biological drugs and the barriers to pulmonary drug delivery. The main challenges in the formulation and inhalation devices are discussed, together with the possible strategies that can be applied to address these challenges. Current clinical developments in inhaled biological drugs for both local and systemic applications are also discussed to provide an insight for further research.

## 1. Introduction

Biological drugs (also known as biologics) are a diverse group of therapeutic agents which are generally large and complex molecules produced through biotechnology [1,2]. Therapeutic peptides and proteins, including antibodies, constitute the largest group of biological drugs and are the focus of this review. Over 25% of novel drugs approved by the FDA between 2015–2019 were biologics covering a broad range of indications [3], including genetic disorders [4], auto-immune diseases [5], cancers [6,7], asthma [8,9] and allergic diseases [10]. The clinical and commercial success of biological drugs is attributed to their high target binding affinity, high specificity of action and desirable safety profile. However, due to their large molecular size and high polarity, the permeability of biologics through the intestinal epithelium is low or even negligible. In addition, enzymatic degradation by peptidases and proteinases in the gastrointestinal tract renders them orally inactive. Biological drugs are therefore currently largely administered by injections [1,11]. Injectable drug therapy however is painful and inconvenient for patients, especially when drugs are used for chronic conditions [12,13]. At least 10% of patients in the world suffer from needle phobia leading to poor compliance [14].

Non-invasive administration routes have been investigated for delivery of biologics, including oral [2,15], buccal [16], nasal [17], inhalation [18] and transdermal routes [19]. In particular, inhalation is an attractive route for delivering biological drugs. The large surface area, extensive vascularisation and high tissue permeability of the lung enable rapid absorption and fast onset of action [20]. Pulmonary delivery also offers the advantage of delivering biologics at high concentrations in the lung. This distribution profile of aerosolised biologics is desirable for the treatment of lung diseases including respiratory infections, lung cancer and asthma [21,22]. Moreover, there is a lower level of proteolytic enzyme activity and minimal first-pass metabolism in the respiratory tract [12]. The application of the lung as an entrance for systemic drug delivery has been investigated for decades. Gänsslen, in 1925, first reported the blood glucose-lowering effect of inhaled insulin (a 51-amino-acid peptide, 5.7 kDa) in five subjects with diabetes [23,24]. In 1989, scientists discovered that human growth hormone (hGH, a 192-amino-acid protein, 22 kDa) was detected in the systemic circulation of rats after intratracheal instillation [25]. These findings demonstrated that the pulmonary epithelial barrier is relatively permeable to macromolecules.

Despite these potential advantages, the development of inhalable biological drugs is challenging. Inhalable formulations need to be rationally designed to achieve appropriate aerodynamic properties for effective lung deposition [18]. The geometry of the airways, humidity, mucociliary clearance and alveolar macrophages are essential in maintaining the sterility of the lung and subsequently pose critical barriers to the therapeutic efficiency of inhaled formulations [26]. In addition, inhaled biologics should exhibit favourable biophysical properties to withstand the stresses encountered during production, aerosolisation and transportation [27]. Moreover, if systemic therapy is desired, inhaled biologics must be able to cross the lung epithelium and reach the blood circulation in sufficient levels in order to exert a therapeutic effect [28].

In this review, the barriers to the development of inhaled biologics are evaluated. The main challenges with respect to formulation and inhalation devices are discussed, together with the possible strategies to overcome these challenges. An overview of the major clinical developments in inhaled biologics for either topical or systemic application are included to provide an insight for future research.

## 2. Structure and Characteristics of Biological Drugs

The majority of the biological drugs on the market have peptidic backbone ranging from small peptides to monoclonal antibodies (mAbs, 150 kDa) [11]. In particular, mAbs are fast-growing biological therapeutics and become a dominant category of recombinant proteins currently used in the clinic [29]. As of October 2020, a total of 17 therapeutic mAbs were approved by the FDA for the treatment of respiratory diseases (Table 1). The major therapeutic mAbs are immunoglobulin G (IgG), which have a long serum half-life of approximately 10–21 days in humans resulting from the physiological recycling mechanism mediated by the neonatal Fc receptor (FcRn) [30,31,32]. Topical delivery of full-length mAbs via inhalation has been investigated in animal models and the therapeutic efficacy in respiratory diseases, including lung cancer [33], asthma [34,35] and pulmonary intoxication [36], are demonstrated. In addition, pulmonary delivery of antibody fragments, such as antigen-binding fragment (Fab) [37], domain antibody (dAb) [38] and single-domain antibody (Nanobody^®^) [27], have also been explored. The development of inhalable antibody fragments is further discussed in Section 4.1.

Biologics are highly labile and prone to degradation when exposed to various types of external stresses, such as elevated temperature, extreme pH, freezing stresses, organic solvent, salt, shear force and light exposure, etc. [28]. Aggregation is the most common and troublesome manifestation of protein instability [39]. It has been observed at all stages of protein product development, leading to reduced bioactivity or increased risk of immunogenicity [40]. Fibrillation is a specific protein aggregation state that can occur naturally in human aetiology. Several neurodegenerative diseases, such as Alzheimer’s disease and Huntington’s disease, are characterised by the formation of protein fibrils (also known as amyloid proteins), an unbranched protein fiber with repeating cross-β sheet structure [41]. Inhaled insulin has been reported to aggregate rapidly and form toxic pulmonary amyloid aggregates at the air-tissue interface. The formation of amyloid deposits causes pulmonary dysfunction after insulin inhalation [42].

This finding suggests that the large surface area and the air-tissue interface in the lungs may promote conformational rearrangement of proteins with misfolding tendency. Therefore, caution should be paid when a protein with amyloidogenic nature is designed to be delivered to the lungs via inhalation.

Proteinaceous drugs with exogenous sequences can be recognised as non-self by the host immune system [43]. Immunogenicity is characterised by the development of anti-drug antibodies (ADAs). The formation of immune complexes between ADAs and the protein therapeutics may accelerate drug clearance, neutralise therapeutic efficacy and induce hypersensitivity reactions [44]. In a study of aerosolised omalizumab in allergic asthmatic patients, one subject developed anti-omalizumab serum IgG and IgA antibodies at day 28 post treatment with reduced serum omalizumab concentration. This finding led to the hypothesis that inhaled mAbs may be more immunogenic than those administered parenterally [35]. A dry powder formulation containing anti-IL-13 mAb fragment, CDP7766, was delivered to the lung of asthmatic patients over a period of 10 days. Nine out of 45 participants were tested positive for anti-CDP7766 antibody after multiple-dose treatment. One subject was considered positive for treatment-associated immunogenicity along with AE of mild and continuous wheezing [45]. All these studies emphasised the importance of determining the degree of immune response in patients following the administration of aerosolised proteins in future studies.

From the delivery point of view, most peptide and protein-based drugs display their pharmacological effect by interacting with cell surface receptors or extracellular ligands, thus the objective of biological drug delivery is to maintain the drug concentration at extracellular sites within the therapeutic window for sufficient period of time [1,46]. Biologics are also associated with a high price tag (from thousands to hundreds of thousands of US dollars per patient per year). Effective biologics delivery systems can potentially lower the administration cost, thus enhancing patient’s and healthcare system’s affordability [47,48]. The unique structure and characteristics of biologics separate them as a special group of therapeutics. The key differences between biological drugs and small-molecule drugs are summarized in Table 2.

## 3. Challenges in Pulmonary Delivery of Biological Drugs

There are several anatomical, physiological and immunological barriers that affect the delivery efficacy of inhaled biologics, including the highly branched structure of the airways, mucociliary clearance, macrophages uptake, pulmonary surfactant, alveolar epithelium permeation and enzymatic metabolism.

### 3.1. Anatomical Barriers

Being able to deliver a sufficient dose of biological drug to the human lung is challenging. The highly branching structure of the lung poses the primary barrier for inhaled particles deposition. The efficiency and the site of deposition of aerosol particles in the lung is significantly affected by their physicochemical properties, including the aerodynamic particle size, shape, charge, hygroscopicity and density [49]. The aerodynamic diameter (D_ae_) is the key parameter that determines the lung deposition efficiency. It is defined as the diameter of a sphere with a unit density that has the same terminal settling velocity in still air as the particle in consideration [50]. In general, particles with D_ae_ between 1 and 5 µm are deposited in the lower respiratory tract, whereas those with size over than 10 µm are deposited in the oropharyngeal region. Particles smaller than 1 µm are exhaled during tidal breathing [50,51].

**Figure 1:**
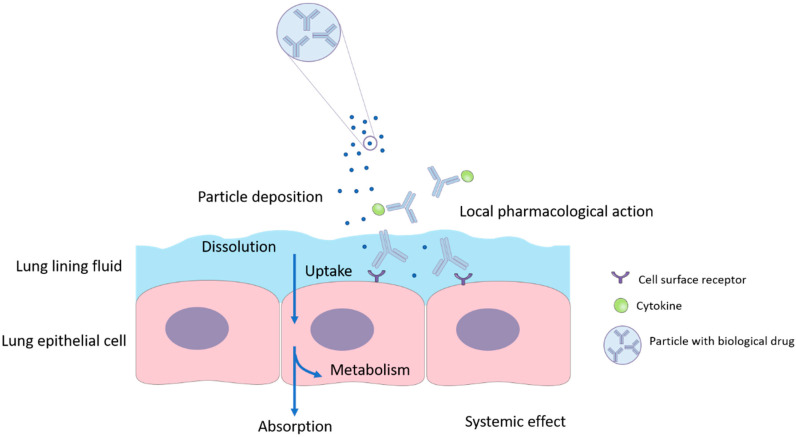
The fate of inhaled biological drugs in the airways upon lung deposition. Following dissolution in the lining fluid, the biological drugs either act on the surface of the epithelial cells, or being taken up by the cells where they are metabolised or absorbed into the systemic circulation.

The optimal site in the lung for the deposition of inhaled biologics is not fully clarified. Aerosol particles must dissolve to release the active drug for subsequent pharmacological action and absorption (Figure 1). However, the amount of fluid in the lung for particle dissolution is limited. The estimated volume of lung fluid is 10 to 30 mL in humans [52]. It is difficult to predict the volume of fluid that an inhaled aerosol particle is exposed to after deposition. In addition, the thicknesses of the lung lining fluid layer is different between central and peripheral lungs. As the airways gradually become smaller in diameter, the lung lining layer becomes thinner until it reaches the alveoli. Particles deposited in the upper airways tend to dissolve quicker because of the larger solid-liquid interface than in alveolar space [53].

The peripheral airways have a significantly larger absorptive surface than the conductive airways, and is considered to be the target region of systemically acting biologics [18]. Epithelial permeability in the upper airway is less favourable because of smaller surface area and reduced regional blood supply [54]. Taking parathyroid hormone 1–34 (PTH) as an example, this polypeptide has a molecular weight of 4118 Da and its bioavailability is correlated with the deposition depth in the respiratory tract. The deeper the deposition in the airways, the larger the extent of PTH absorption [55]. It appears to be important for small peptides to deposit in the alveolar space rather than the upper airways for optimal absorption [24]. On the other hand, if the biological drugs are transported by an active mechanism, their absorption is correlated with the expression pattern of the receptors in the respiratory tract. A good example is FcRn, an IgG binding receptor that plays an important role in the recycling of IgG in the bloodstream [56]. This receptor is also expressed in the lung, with expression restricted to the epithelial cells of upper airways and alveolar macrophage in humans and macaques [21,56]. Thus, the ideal place for the absorption of IgG might be in the upper and central airways where receptor-mediated transcytosis takes places. The size of aerosolised particles with Fc-fusion proteins (with mass median aerodynamic diameter of 4.1 µm) was designed to target the upper and central respiratory tract, where the expression of FcRn is prominent [57].

### 3.2. Mucociliary and Macrophage Clearance

Mucociliary clearance is a dominant defense mechanism in the upper conduction airways against potentially harmful particles [58]. It exerts its function by integrative activity of non-cellular (the mucus layer) and cellular elements (the cilia and secretory cells) [53,59]. In healthy subjects, inhaled insoluble particles deposited in the airways are swept up and cleared from the respiratory system within 24 h [24]. Anti-IL-17A antibody fragment was delivered to the lung of mice by intratracheal instillation. Up to 29% of anti-IL-17A-Fab’ was found in the stomach at 4 h post pulmonary delivery, suggesting a substantial proportion of Fab’ fragment was cleared by mucocilliary clearance [60]. Furthermore, since peptides and proteins are highly charged and hydrophilic, mucus components tend to interact with them and retard their diffusion, thereby limiting drug absorption [61]. For instance, there is an electrostatic repulsion between the negatively charged protein molecules and mucin fibers [62], subsequently preventing them from a close contact with the lung epithelium [12]. It is reported that Fc fragment of antibodies is the major moiety binding with mucins. The number of Fc fragment within an antibody controls the diffusion rate of antibodies in mucus. Proteins such as IgG (with one Fc fragment) permeates through mucus as fast as through saline, whereas the diffusion of IgM (with several Fc fragments) is significantly hindered [63].

In the peripheral lungs, macrophage uptake plays a significant role in alveolar clearance of inhaled biological drugs [64]. A confocal imaging study showed that a large amount of hGH was taken up by alveolar macrophage 1 h post intratracheal delivery in rats and retained at least 4 h [65]. After being phagocytosed, inhaled particles could be either degraded by intracellular enzymatic lysosomal system; transported to the lymphatic system; or migrated along the ciliated airways and removed from the respiratory tract by mucociliary clearance [28]. The uptake by alveolar macrophages is size-dependent. Particles with geometric diameter range between 1–2 μm are easily phagocytosed by macrophages (15–22 μm in diameter) and those with smaller size are taken up less effectively [24]. Depletion of alveolar macrophage by dichloromethylene diphosphonate containing liposome improved the systemic absorption of IgG and human chorionic gonadotropin (hCG, 39.5 kDa) in severalfold scale following intratracheal instillation in rats [66]. The degradative pathway of alveolar macrophage competes with the epithelial transport in airway lumen. Large proteins such as IgG with slow absorption rate stay in the alveolar space for hours, which provides sufficient residence time for macrophage uptake. In contrary, systemic absorption of small peptides and proteins, e.g., insulin [66] and hGH [64], was less affective by macrophage depletion as they transport rapidly through the alveolar epithelium.

### 3.3. Pulmonary Surfactant

Pulmonary surfactant (PS) is a continuous liquid layer spreading from the distal to the proximal lungs. It is the first contact for aerosol particles deposited in the airways [67] and the fate of particles depend on their interaction with the PS layer. The composition of PS includes phospholipids (~92%, by mass) and surfactant proteins (~8%) [68]. Large proteins may interact with PS components, triggering aggregation and subsequent macrophage degradation [69]. PS was conventionally considered as a barrier for drug delivery in the peripheral lung, where mucus is absent in healthy condition [70]. On the other hand, PS has been investigated as a carrier to promote the transportation of drugs in the airways [67]. It was shown that natural PS and its most abundant phospholipids, dipalmitoylphosphatidylcholine (DPPC), are potential absorption enhancer of inhaled peptides and proteins [71]. Addition of DPPC in the formulation has been shown to promote the absorption of insulin [72], parathyroid hormone [55] and hGH [65] in the lungs of rats. The absorption enhancing effect of PS probably was mediated by opening the tight junction of the cell monolayer to accelerate the paracellular transport of hydrophilic protein molecules [55]. Little is known about the interactions between surfactant biomolecules and inhaled biologics at a molecular level and this gap in knowledge necessitates further research.

### 3.4. Airway Epithelium

Inhalation has been investigated for local or systemic delivery of biological drugs. For locally-acting drugs, inhaled formulation needs to solubilise in the pulmonary lung fluid to interact with the mucus and target cells in the airways in order to elicit the pharmacological effect. For systemically-acting biological drugs, the drug molecules must be able to gain access to the systemic circulation in addition to proper lung deposition and dissolution [18]. The pulmonary epithelium is the primary barrier for the transportation of protein drugs to the bloodstream [73]. Transportation of biological drugs across respiratory epithelium is size-dependent. Small soluble peptides and proteins with molecular weight below 40 kDa are rapidly detected in the bloodstream, whereas those with molecular weight above 40 kDa are slowly absorbed in the lung over hours to days [24,26,69]. Small peptides are rapidly absorbed, but in parallel are subject to substantial metabolism in the airways [52]. Larger proteins, e.g., antibody fragments [45,60] and mAbs [21,22], are absorbed very slowly with limited bioavailability.

**Figure 2:**
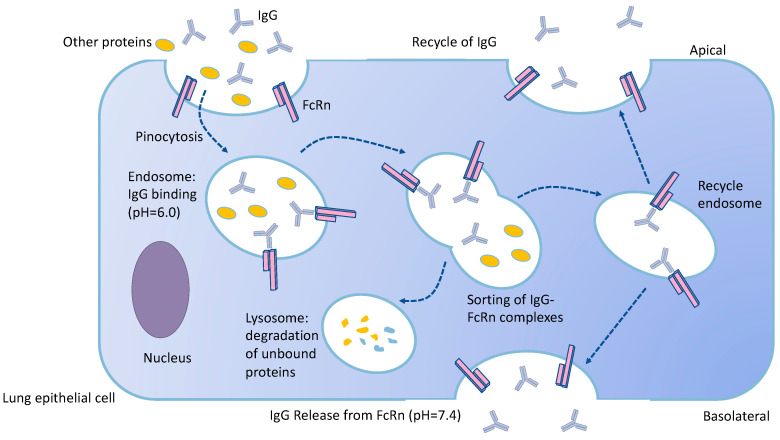
Transport and recycle of IgG in the lung epithelium by the FcRn-mediated pathway. IgG is taken up into FcRn-expressing epithelial cells via non-specific fluid-phase pinocytosis. IgG then binds to the transport receptor, FcRn, which is mainly distributed within the acidic endosomal vesicles in the cell. The Fc receptor binds to IgG with high affinity at low pH but shows no affinity at physiologic pH. FcRn with bound IgG is sorted into the sorting endosome, which either recycle or transcytose to the plasma membrane. Nonprotected IgG and proteins proceed to lysosomal proteolytic degradation. The near-neutral pH results in dissociation and release of IgG from FcRn into the extracellular fluid.

There are two possible mechanisms for biologics to cross the pulmonary epithelium, namely paracellular transport and transcytosis [74]. Peptides and proteins without specific receptors in the lung epithelial cells are transported by paracellular pathway or non-specific pinocytosis [75]. A number of biological drugs are actively permeate the alveolar epithelium by receptor-medicated transcytosis, e.g., albumin [76], alpha-1 antitrypsin (AAT) [77] and transferrin [73,78]. Both pathways are involved to mediate the transportation of insulin, although the contribution of each pathway is unknown [75,79]. FcRn, a IgG-binding receptor, facilitates the pulmonary absorption of Fc-fusion transportation via FcRn-mediated transcytosis in the upper and central respiratory tract [57,80,81,82]. FcRn also plays a key role in the recycling of mAbs in the airways (Figure 2). The mean residence time of cetuximab in the lung tissue of FcRn-wild-type (WT) mice was 10 times longer than that in FcRn-knockout (KO) animals, suggesting FcRn may contribute to the relocation of mAbs from lung tissue to the lung lumen [21].

Two major cell types are found in the alveolar epithelium, namely type I and type II pneumocytes, joined by tight junctions. Over 90% of the alveolar surface is covered by type I cells, while type II cells make up to 5–10% of the surface. Subcellular morphology study revealed that endocytic vesicles are present in type I cells [54], which are likely the major cell types for drug absorption in the lung [83]. The relative contribution of type I and type II pneumocytes in overall transportation of peptides and proteins remain to be determined. It was reported that albumin (66 kDa) is predominantly taken up by receptor-mediated endocytosis in alveolar type II cells. Although type II cells occupy only a small proportion of surface area in alveoli, the uptake of albumin in type II cells is higher than type I cells [84]. Given the role of endocytosis/transcytosis in transepithelial trafficking of inhaled biologics, identification of specific receptors involved in the cellular uptake and understanding the transport mechanism are important for the development of efficient pulmonary delivery strategies for biological drugs [79].

### 3.5. Metabolism

Many types of protease and peptidase are found in the airspace and epithelial cells [52]. Alveolar macrophages and other inflammatory cells (e.g., neutrophils) are the sources of proteases. The extent to which protein molecules are metabolised is unclear. The level of proteases is higher in the airways in inflamed lung, thus the efficacy of aerosolised proteins and peptides may be impaired [26]. Enzymatic hydrolysis of small natural peptides (less than 3 kDa) is high unless they are chemically modified to block the activity of peptidase. As the molecular weight increases, proteins with greater tertiary and quaternary structure could inhibit peptidase hydrolysis [24,69]. In general, proteins with molecular weights between 6 and 50 kDa are relatively resistant to most peptidases and have good bioavailability upon inhalation [69]. Formulations that retard enzymatic degradation or promote drug absorption can increase bioavailability [55]. Addition of protease inhibitors to the formulation was shown to improve the pulmonary absorption and bioavailability of peptides and proteins. Given the potential toxicity, safety is a major concern of formulations with protease inhibitors, especially in chronic use [20].

## 4. Strategies for Inhaled Delivery of Biological Drugs

Various approaches have been applied to modify the structure of biological drugs to enhance pulmonary absorption or maintain in vivo stability, such as antibody fragment development, Fc engineering and PEGylation. A brief summary of inhaled biological drugs investigated preclinically for pulmonary delivery is shown in Table 3.

### 4.1. Antibody Fragments

**Figure 3:**
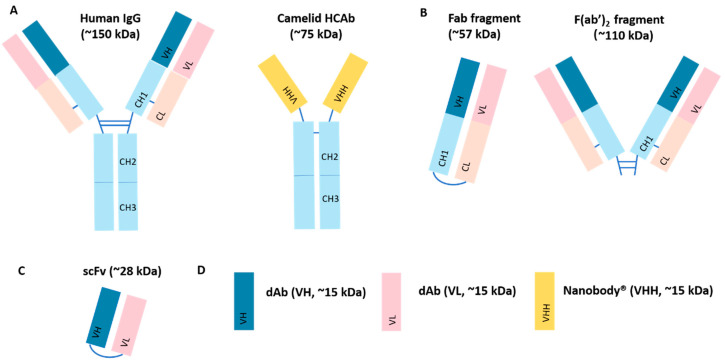
Schematic representation of different antibody fragments investigated for pulmonary delivery. (**A**) Conventional antibodies from mammals have Y-shape structure composed of two identical heavy and light chains. Each chain contains constant regions (CH1, CH2, CH3 and CL) and variable regions (VH and VL) that are held together by disulfide bonds. Members of the Camelidae family naturally produce a unique type of immunoglobulin that is devoid of light chains. These heavy chain-only antibodies (HCAbs) are formed by a single variable domain (VHH) and two constant domains; (**B**) Fab is an antibody fragment that can bind to antigens without Fc region; F(ab’)2 contains two Fab fragments linked together by disulfide bonds; (**C**) Single-chain fragment variable (scFv) is composed of variable domains of the heavy (VH) and light chains (VL) of human IgG, connected by a peptide linker; (**D**) Domain antibodies (dAbs) are antibody fragments derived from the variable domains of either the heavy or light chain of human IgG. Nanobody^®^ is the recombinant single-domain antibody derived from the HCAbs of Camelidae.

Structural simplification is a strategy to improve their in vivo delivery efficacy of inhaled biological drugs. Smaller fragments of antibodies that are either expressed by bacterial or yeast cultures [85], or obtained by the digestion of full-length antibodies with proteolytic enzymes [86], have emerged as a separate class of protein-based therapeutics. Antibody fragments offer the advantages of enhanced tissue penetration, easy and inexpensive production. Given the lack of an Fc region, antibody fragments are subject to rapid degradation with short serum half-life in vivo [87]. Several antibody fragments are developed and investigated for pulmonary delivery (Figure 3). The pharmacologically active antigen-binding region is preserved in the Fab, which has been delivered to the lungs via inhalation to neutralise inflammatory cytokines. The efficacy of nebulised anti-IL-13 Fab fragments (CA154_582 [88] and CDP7766 [89], UCB Pharma, Brussels, Belgium) were assessed in experimental models of allergic asthma (mouse and cynomolgus macaque). Inhaled anti-IL-13 Fab’ was well-tolerated and markedly suppressed airway inflammation in mice, whereas systemic administration of Fab’ failed to show these effects, probably due to its short half-life (4 h) in the circulation [88]. CDP7766 was subsequently formulated into a dry powder for inhalation and investigated in human subjects (NCT02473939). Single or multiple inhaled dose were well tolerated in healthy and asthmatic subjects respectively [45].

Domain antibodies (dAbs) are antibody fragments derived from the variable domains of either the heavy or light chain of human IgG. These are the smallest functional unit of human antibodies with antigen-binding activity [90]. The investigation of pulmonary delivery of dAbs was focused on the prophylaxis and treatment of acute lung injury (ALI). Specific inhibition the signaling of tumour necrosis factor (TNF) receptor-1 (TNFR1, p55) [38,91] via inhaled dAbs (GSK1995057, GSK, Brentford, UK) significantly reduced the airway inflammation in animal (mice and cynomolgus monkeys) and human models of ALI (NCT01587807). In contrast, intratracheal administration of conventional anti-TNF mAbs was inefficient. The treatment efficacy of the dAb probably was attributed to the smaller molecular size (~12 kDa), leading to better tissue penetration and favourable biodistribution [91]. Unexpectedly, in clinical trials of intravenous GSK1995057, a novel human anti-VH (HAVH) was identified in around 50% of healthy human subjects. The binding of autoantibody to framework region of GSK1995057 induced cytokine release and subsequently increased the risk of infusion reaction [92]. Therefore, an antibody derivative (GSK2862277) with reduced binding frequency to HAVH autoantibodies was produced and investigated in clinical trials. A single dose of inhaled GSK2862277 was well tolerated in patients with risk of ALI [93].

Nanobodies^®^ (a registered trademark of Ablynx, Ghent, Belgium) are recombinant single-domain antibodies derived from the heavy chain-only antibodies (HCAbs) of Camelidae [94]. Nanobodies^®^ combines the advantages of small-molecule drugs such as smaller size, good stability and ease of production, with the characteristics of high selectivity and affinity of conventional antibodies [95]. These biophysical characteristics make Nanobodies^®^ especially relevant to antiviral therapeutics, e.g., RSV infection [96,97], influenza virus infection [98] and Middle East respiratory syndrome coronavirus infection (MERS-CoV) [99,100,101,102] and COVID-19 [103,104]. ALX-0171 is a 42-kDa trimeric Nanobody^®^ designed to target the RSV surface fusion (F) protein [96]. It inhibits the release of RSV from the apical surface in cell culture [105]. Nebulised ALX-0171 was well-tolerated, dramatically reduced viral loads and lung lesions in RSV infection models of cotton rats [27,96] and neonatal lambs (lambs and human infants share numerous anatomical and physiological similarities) [97,106]. In light of these findings, two Phase II studies were initiated to evaluate the safety and efficacy of inhaled ALX-0171. A dose-dependent antiviral effect was observed in hospitalised infants and young children with RSV infection, but without consistent improvement of clinical outcome (NCT02979431) [107]. The second clinical trial in Japanese children was discontinued due to the lack of clinical efficacy (NCT03418571). Recently, the COVID-19 pandemic encourages the exploration of novel treatment strategies against viral infection. The cellular entry of SARS-CoV-2 is achieved by binding of viral spike glycoprotein to the angiotensin converting enzyme 2 (ACE2) receptor on the surface of host cell. Nanobodies, e.g., Nb11-59 (15 kDa, Novamab Biopharmaceuticals, Shanghai, China) [103] and Ty1 (12.8 kDa, Karolinska Institutet, Stockholm, Sweden) [108], are developed to target the receptor binding domain (RBD) of SARS-CoV-2 spike glycoprotein, blocking the virus entry. These nanobodies exhibited potent viral neutralising activity in vitro and remained stable during nebulisation [103]. Inhaled nanobodies are promising antiviral therapy against COVID-19 viral infection.

### 4.2. PEGylation

**Figure 4:**
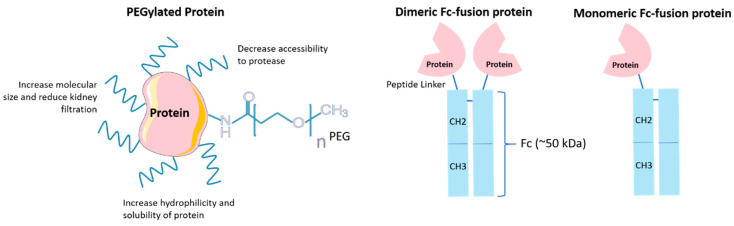
Structure of PEGylated protein and Fc-fusion protein. PEGylation is a chemical process involving the attachment of polyethylene glycol (PEG) chains to a molecule of interest through covalent binding or non-covalent complexation; Fc-fusion proteins are composed of the Fc domain of IgG genetically linked to a peptide or protein of interest. The first-generation Fc-fusion proteins are dimeric wherein an effector molecule is fused with each arm of the Fc fragment. The second-generation Fc-fusion proteins are monomeric wherein a single effector molecule is conjugated to one arm of the Fc fragment.

Enzymatic degradation and rapid lung clearance are two significant challenges faced by inhaled biologic therapies in the treatment of respiratory diseases. Prolonging the retention of inhaled biomolecules could be beneficial due to an altered pharmacokinetic profile (longer duration of local effects) and reduced dosing frequency [115]. Conjugation of one or more hydrophilic polyethylene glycol (PEG) chains to peptides and proteins can increase the molecular mass and provide shielding effect to the conjugated molecules. PEGylation protects proteins from renal clearance and proteolytic enzyme degradation, subsequently prolongs the protein residency in the body [116] (Figure 4). PEGylation has been demonstrated to be an effective method to extend the retention time of therapeutic proteins in the lung, including human alpha1 proteinase inhibitor (α_1_-PI, also known as alpha-1-antitrypsin, AAT) [113], IFNα [114] and antibody fragments [37,60,111]. The PEGylated Fab fragment, anti-IL-17A PEG40-F(ab′)_2_, has display improved efficacy in reducing biomarkers and lung inflammation in a murine model of allergic asthma. PEGylation enhances the stability of antibody fragments in the airways by mucoadhesion, avoidance of alveolar macrophage uptake [111] and decreased transepithelial transport to the bloodstream [60]. The residence time of PEGylated antibody fragments was highly dependent on the deposition site in the respiratory tract; the deeper the deposition, the longer the residency. The prolonged residency of proteins in the deep lungs is attributed to the decreased mucociliary clearance activity from the central to distal airways [60]. The number and size of the PEG chains, as well as the site of PEGylation are key parameters in determining the bioactivity and pharmacokinetic properties of conjugated proteins [115]. Optimisation of PEGylation is required in each delivery system to confine exposure of the biologics to the lungs so as to minimise systemic adverse effects and repeated administration [114].

### 4.3. Fc Engineering

Proteinaceous molecules can be linked to the Fc fragment of IgG to produce Fc-conjugated or Fc-fusion proteins [56]. These hybrid biomacromolecules have prolonged plasma half-life because of FcRn-mediated recycling in the blood vessel endothelium [80]. The FcRn-mediated transportation across the respiratory epithelial barrier was initially demonstrated by pulmonary delivery of erythropoietin Fc-fusion protein (EpoFc, ~112 kDa) in cynomolgus monkeys [57]. Epo is a glycoprotein hormone that triggers the formation of red blood cells. Intravenous or subcutaneous injections are normally required for chronic treatment. EpoFc translocated across the lung epithelium and remained bioactive. The bioavailability of inhaled monomeric EpoFc was comparable to that of a subcutaneously administered unconjugated Epo. The level of circulating reticulocytes increased 5–7 days after single inhaled dose of EpoFc. EpoFc monomer displayed higher receptor affinity and pulmonary uptake compared to EpoFc dimer (Figure 4). The delivery efficacy of inhaled EpoFc was further studied in healthy subjects in a Phase I clinical trial. A dose-dependent concentration of fusion proteins and an increase of reticulocytes were observed in the blood circulation [117]. The Fc fragment of human IgG1 has flexibility for conjugation with other proteins to improve their transepithelial transport or half-life in the airways, including interferon beta protein (IFNβ) [82] and follicle-stimulating hormone (FSH) [81]. Together, these results illustrate the potential of pulmonary delivery of bioactive Fc-fusion protein as a more acceptable alternative to the parenteral dosage forms.

## 5. Inhalation Technology

Successful delivery of inhaled biological drugs requires not only a proper formulation but also an inhaler device to generate drug aerosols. Due to the complex structure of biological drugs, the aerosolisation process is required to produce aerosol particles with aerodynamic properties suitable for inhalation, and at the same time preserve the physical integrity and potency of drug molecules. Moreover, patient compliance and acceptance are critical as part of disease management in inhalation therapy. An ideal inhalation system should be user-friendly, convenient to use, portable and inexpensive [49].

### 5.1. Nebulisation

Therapeutic biologics currently on the market are predominantly formulated as suspensions or aqueous solutions either in a ready-to use form or as a lyophilised powder for reconstitution [118]. Investigational biological drugs are initially prepared in a liquid form in early development stage to avoid extra process steps such as drying [119,120] (Table 3). Around 75% of the inhaled protein formulations in clinical research were prepared as liquids for nebulisation [121]. Nebulisation has drawbacks such as low delivery efficiency, long administration time, poor reproducibility and relatively high costs of device maintenance [49]. Nevertheless, it also provides several considerable advantages, including the avoidance of drying process during manufacture and suitability for patients of different ages and stages of illness [119]. Ultrasonic nebulisers, jet nebulisers and vibrating-mesh nebulisers are the three major types of nebulisers used in the management of lung diseases. Protein aggregation and unfolding at the air-liquid interface are especially challenging during aerosolisation process in ultrasound and jet nebulisers [119], while vibrating mesh nebulisers are more suitable for biological drugs as they do not produce heat and are less likely to denature the molecules [22,36]. The drug and the device should be optimised together to achieve desirable aerosol performance and retain the molecular integrity of biologics [122]. Nebulisation of Nanobody^®^ ALX-0171 via jet nebuliser triggered significant protein multimerisation and aggregation, which were barely detected in vibrating mesh nebuliser [27].

There have been significant advances in aerosol delivery technologies in the last two decades [123]. Small, portable nebulisers with fast delivery rates have emerged. Dornase alfa (Pulmozyme^®^, Genentech, South San Francisco, CA, USA) is a recombinant human deoxyribonuclease I (rhDNase) for the management of cystic fibrosis (CF) by inhalation. Only jet nebuliser/air compressor combinations were recommended to deliver the drugs after initial regulatory approval in 1993. In 2015, The eRapid™ nebuliser system (PARI Respiratory Equipment, Midlothian VA) was approved as the first electronic nebuliser by the FDA to deliver Dornase alfa for CF patients. The eRapid™ nebuliser system is smaller, lighter and quieter than conventional jet nebulisers. It also provides advantages of shorter nebulisation time and higher patient preference [124]. Due to the high cost of biologics, an efficient device is important to deliver a high payload to the airways. Smart nebulisers that provide accurate dosing independent of lung function are in development. Both I-neb adaptive aerosol delivery (AAD) system (Philips Respironics, Murrysville, PA, USA) and AKITA^2^ APIXNEB^®^ (Vectura, Chippenham, UK) are breath-controlled devices used to enhance the delivery of AAT in CF. They are electronically regulated inhalation systems coupling an electronic control unit with a vibrating mesh nebuliser. These devices control the inhalation by the patient’s breathing patterns and generate inhalation parameters unique to the individual [125,126]. AKITA^2^ APIXNEB^®^ inhalation system delivered human α_1_-PI (Prolastin^®^-C, Grifols, Barcelona, Spain) to the lungs of CF patients and achieved a high overall lung deposition (around 70% of the drug loaded into the nebuliser), irrespective of the severity of lung function impairment [126]. Additionally, AKITA^2^ APIXNEB^®^ system was used to aerosolise a couple of therapeutic proteins for pulmonary delivery, e.g., recombinant granulocyte-macrophage colony-stimulating factor (rGM-CSF) [127], Dornase alfa [128] and ALX-0171 Nanobody^®^ [96]_._

### 5.2. Pressurised Metred-Dose Inhalers (pMDIs)

pMDIs use propellants to generate aerosol for inhalation. Drugs are either dissolved or suspended in a single propellant or propellant mixture together with excipients such as co-solvents and surfactants. To date, there is no approved pMDI product for inhaled biologics therapy [129]. Nevertheless, biologics formulations for pMDIs are being investigated. Proteins and peptides are generally hydrophilic and therefore are poorly soluble in non-polar hydrofluoroalkane (HFA) propellants [130]. Biologics are formulated as suspension rather than solution in pMDI formulation, which substantially limits the formulation possibility. Poor solubility of biologics in the propellants also limits the dose range that can be delivered per actuation [131]. In addition, only a few proteins (e.g., lysozyme) was demonstrated to have reasonable stability when formulated as a suspension in pMDI [132]. A major concern is the denaturation of proteins and peptides when they interact with the propellants [20]. To enhance the stability, therapeutic proteins could be incorporated in a particulate carrier to suspend in a propellant. Co-spray drying proteins (e.g., lysozyme, catalase and bovine serum albumin) with polyvinyl alcohol (PVA) [130] and sodium carboxymethylcellulose (NaCMC) [133] can improve the physical stability of protein particles in surfactant-free HFA propellent. Nanoparticles (NPs) were also reported to improve the stability of proteins such as insulin in pMDI formulation [134]. NPs (fabricated by a bottom-up process) containing lysozyme [135] or thymopentin [136] were readily dispersed in HFA 134a to form a stable suspension with good re-dispersibility and bioactivity. These studies highlight the potential for biologics delivery using pMDI formulations.

### 5.3. Dry Powder Inhalers (DPIs)

DPIs are propellant-free, portable and easy-to-use inhalation device [137]. When considering DPIs for biologics, it is important to identify a suitable drying method that can produce particles with good aerosol property and preserve the integrity of the biological drugs. While milling is the most commonly used technique to generate inhalable dry powders, this approach is not suitable for fragile molecules that are prone to degradation [112]. Spray drying (SD) is a single-step, continuous and scalable particle processing technique in the pharmaceutical industry [49]. It has been employed to produce inhalable dried powders of biologics, such as insulin [138], DNase [139], anti-IgE mAb [140], hGH [65], IgG1 [141] and infliximab [34]. During SD, liquid feed is atomised into a hot drying gas where the solvent is evaporated to produce dried particles. Thermal stress and high shear force may denature and hence inactivate biological molecules [49]. Stabilising excipients can be added to maintain the structural stability and bioactivity of biologics. Sugars are commonly used as protective excipients in biological formulations, including disaccharides (e.g., trehalose) [34]; alcohol sugars (e.g., mannitol, sorbitol) [141]; and oligosaccharides (e.g., cyclodextrins) [142]. The application of lactose is limited in protein formulations because of the Maillard reaction. Therefore, non-reducing sugars are preferred in the context of protein stabilisation [143]. Due to the lack of safety data, the list of approved inactive ingredients for the respiratory route is exceptionally short, necessitating safety assessments of new excipients alone and in the final formulations [49,120]. Excipient-free peptide dry powder inhalation has been investigated recently [144,145]. This approach negates any concerns about safety and tolerability of the excipients in the formulation.

Spray freeze drying (SFD) is another drying technique that combines SD and freeze drying. A liquid is first atomised into a cryogen (usually liquid nitrogen), followed by lyophilisation [146]. This method is less well-established in pharmaceutical industry compared to SD due to scale-up difficulties [147]. However, the SFD particles usually exhibit porous structure with good aerosol performance, making them particularly attractive for inhalation [146]. The avoidance of high temperature also makes SFD suitable to generate inhalable dry powders of thermolabile materials like vaccines with retained potency and immunogenicity [112,148]. Various platforms were developed in the past two decades to prepare engineered protein microspheres for inhalation, such as Technosphere^®^ [149], PROMAXX^®^ [150] and particle replication in non-wetting templates (PRINT^®^) [151]. These techniques are compatible with model proteins and yield particles in a size range suitable for inhalation, with potential to apply to other therapeutic proteins.

A concern associated with breath-actuated DPIs is that patients are incapable to generate sufficient inspiration force to disperse the powder into aerosol for efficient lung deposition. Active inhaler has been developed to overcome this shortcomings [152]. Considering the inhaled insulin product Exubera^®^ as an example, the inhaler uses compressed air to disperse powder into a spacer reservoir prior to inhalation. However, the device was cumbersome and heavy that significantly affected its acceptability, which was one of the major reasons leading to its commercial failure and eventual withdrawal from the market [153]. Given to the technological barrier of DPIs design, inhaled biologics are predominantly formulated as an liquid formulation in the product development phase. To our knowledge, there are only three DPIs, F1P (multidose, Vectura, Chippenham, UK) [45], Cyclohaler^®^ (single dose, PB Pharma GmbH, Meerbusch, Germany) [154] and Concept1 (single dose, Novartis, Basel, Switzerland) (NCT04410523) have been tested in clinical trials to deliver therapeutic antibody fragments and proteins to the lungs.

## 6. Clinical Developments

A number of inhaled biologic formulations are undergoing clinical developments for the treatment of both respiratory diseases (e.g., CF, respiratory virus infection and asthma) and systemic disorders (e.g., diabetes).

### 6.1. Inhaled Therapeutic Proteins and Peptides

Inhaled proteins have been investigated as a therapy of inherited disorders, including CF [155], alpha-1 antitrypsin deficiency (AATD) [156,157] and pulmonary alveolar proteinosis (PAP) (NCT01511068 and NCT02243228). CF is characterised by excessive mucus secretion, recurrent airway infection and chronic neutrophilic inflammation in the respiratory tract [158]. Dornase alfa, a 37-kDa rhDNase I, is the first inhaled protein approved by the FDA for the treatment of CF. It acts as a mucolytic agent to reduce the viscosity of mucus in the airway [121]. Daily aerosolised rhDNase I leads to improvement in lung function and reduction the incidence of exacerbations in CF patients [159]. Dornase alfa is a classic example of an inhalable therapeutic protein that was successfully made into the market for respiratory diseases. However, the activity of DNase I is substantially inhibited by actin, a protein found in high amount in CF sputum. Alidornase alfa (PRX-110 or AIR DNase™, Protalix, Carmiel, Israel), a chemically modified plant cell-derived DNase I, was developed to resist the inhibition activity of actin. The safety and efficacy of inhaled Alidornase alfa was investigated in a Phase II switchover trial in CF patients treated with Dornase alfa before (NCT02605590). Interim analysis indicated that Alidornase alfa was well tolerated and potentially improved the lung function in CF patients [155].

Excessive release of neutrophil elastase (NE) mediated by the recruitment of neutrophil in the airways of CF causes subsequent lung tissue damage. AAT, a serine protease inhibitor, is a natural antagonist of NE. The normal balance of NE/AAT is disturbed in CF, resulting in accumulation of NE in the lungs [160]. Inhaled AAT may provide a protective effect and restore the protease and anti-protease homeostasis in the respiratory tract. Alpha-1 HC (Grifols, Barcelona, Spain) and Kamada-AAT (Kamada, Rehovot, Israel) are two inhaled AAT formulations currently assessed in Phase II clinical studies. Inhaled AAT therapies demonstrated acceptable safety and tolerability in CF patients. A reduction of neutrophils and NE in sputum were found in patients treated with Kamada-AAT [160], while nebulised alpha-1 HC failed to induce a clinically meaningful improvement in patient’s lung function and sputum biomarkers [158]. Since the number of patients in these trials was small (41 and 21 subjects, respectively), further studies with longer treatment periods and larger sample sizes are warranted.

AATD is a hereditary disease that causes emphysaema. It occurs in up to 3% of chronic obstructive pulmonary disease (COPD) cases. AATD patients have a higher risk of developing early onset of COPD after cigarette exposure [156,157]. Weekly intravenous infusion of AAT (Prolastin^®^-C liquid) is indicated for the chronic treatment of AATD lung diseases. Inhaled AAT therefore has been studied as a non-invasive substitution therapy of AATD [13]. In a phase II study, Kamada-API (52 kDa) was delivered to AATD individuals once or twice daily over 12 weeks by inhalation. Aerosolised AAT decreased neutrophil levels and NE concentrations in the airways. Furthermore, AAT was detected in the plasma of all the study subjects treated with inhaled AAT, confirming the transportation of AAT from the alveolar space to the bloodstream [161]. A phase III clinical trial has been initiated in 2019 to evaluate the safety and efficacy of inhaled Kamada-API in AATD patients (NCT04204252).

Inhaled proteins therapy also aimed at treating asthma. A subgroup of asthmatic patients (~50%) have eosinophilic airway inflammation. The activation and recruitment of eosinophils in the airways are driven by type 2 T helper cell (Th2)-specific cytokines, e.g., IL 4, 5 and 13 [162]. Pitrakinra (Bayer, Wuppertal, Germany) [163] and AZD1402/PRS-060 (AstraZeneca, Cambridge, UK and Pieris Pharmaceuticals, Boston, MA, USA) [164] are inhaled IL-4 muteins targeting IL-4 receptor alpha (IL-4Rα), which were developed as novel treatments of asthma. In a Phase II study, inhaled pitrakinra attenuated the late phase asthmatic response and airway inflammation in asthmatic subjects after allergen challenge [163]. AZD1402/PRS-060 is another IL-4Rα antagonist with higher inhibitory effect to IL-4Rα than Pitrakinra in vitro [164]. Single inhaled dose was well tolerated in healthy volunteers [165]. A multiple ascending dose study of AZD1402/PRS-060 in adult patients with mild asthma is ongoing (NCT03574805).

Pulmonary delivery of proteins have been investigated to fight against respiratory viral infections. DAS181 (Fludase^®^, Ansun BioPharma, San Diego, CA, USA) is a sialidase fusion protein, which attenuates influenza virus infection by inhibiting the attachment of virus to the lung epithelium [166]. DAS181 was formulated as an inhalable dry powder to target lung epithelial cells. Inhaled DAS181 was safe and significantly decreased viral load in patients infected with influenza [154,167]. SNG001 (Synairgen, Southampton, UK) is an inhaled formulation containing interferon beta (IFN-β), a natural protein controls viral infection in the body. Phase II studies showed that inhaled SNG001 boosted antiviral defense in both asthmatic [168] and COPD subjects [169], and potentially improved the asthmatic symptom induced by cold or flu infection [170]. Since the anti-viral effect of DAS181 and SNG001 is host-directed, they offer a new strategy to target other viruses and strains. Indeed, SNG001 [171] and DAS181 (NCT04324489 and NCT03808922) are currently being explored for the treatment of COVID-19 infection. Positive results were reported in hospitalised COVID-19 patients treated with nebulized SNG001 [172]. Inhaled protein therapeutics might be a potential treatment option for patient with severe COVID-19. Additionally, topical delivery of proteins via inhalation was studied in a range of respiratory diseases, e.g., lung cancer [173], lung bacterial infection [174] and acute plastic bronchitis [175]. A selection of biologics in clinical development and marketed products for topical lung delivery are summarised in Table 4.

The lung can serve as a portal for the entry of biological drugs intended for the systemic circulation, which is exemplified by the development of inhaled insulin [176]. The first inhalable insulin product, Exubera^®^, developed collaboratively by Pfizer (New York, NY, USA) and Nektar Therapeutics (San Carlos, CA, USA), received approval from the FDA in 2006 for the treatment of types 1 and 2 diabetes [138]. The stabilisation of spray-dried insulin peptide to retain its biological activity in the powder form was pivotal in the success of Exubera^®^. Since Exubera^®^ was rapid-acting, longer-acting insulin would still be required for certain patients. This had a negative impact on the cost-effectiveness of Exubera^®^ compared with other existing treatment options. Furthermore, with Exubera^®^ the insulin dose was measured in milligrammes, which differs from the conventional international units used in other insulin products. This created inconvenience, and more importantly, raised apprehension over dose conversions and prescribing errors. Another safety concern was the risk of lung cancer with Exubera^®^ use [177]. Despite being a global breakthrough in biotherapeutics, Exubera^®^ was eventually withdrawn from the market in 2007 because of poor sales [178]. The exit of Exubera^®^ persuaded most companies to discontinue their development programmes for inhaled insulin, with the exception of MannKind (Valencia, CA, USA), who later received FDA approval in 2014 for Afrezza^®^, another DPI like Exubera^®^. Afrezza^®^ is marketed as an ultra-rapid-acting inhaled insulin indicated to improve the glycaemic control for adult patients with type 1 or 2 diabetes [153]. Compared to its predecessor, Afrezza^®^ is not as unwieldy and offers better flexibility in dosing [179]. The examples of Exubera^®^ and Afrezza^®^ illustrate that technical advance and patient acceptance are equally important for market success of an inhaled biologic product.

### 6.2. Inhaled mAbs and Antibody Fragments

The therapeutic potential of inhaled mAbs and their derivatives has been evaluated in a number of clinical trials. One study tested the efficacy of omalizumab self-administered via nebulisation in patients with mild allergic asthma. Omalizumab was detected in the serum, albeit no remarkable changes to serum IgE levels were observed and allergen-induced airway responses remained uninhibited. The inefficacy was postulated to be attributed to the inadequate omalizumab concentrations attained in the lung tissue encompassing IgE effector cells to neutralise IgE. Furthermore, one participant developed anti-omalizumab antibodies, sparking concerns that inhaled mAbs might be more immunogenic compared with those given via injection [35].

Several inhaled antibody fragments have entered clinical-trial stage for the treatment of acute lung injury [38,93] and asthma [45]. In a Phase I clinical trial, healthy human subjects were received a single dose of nebulised GSK1995057, a dAb directed against TNFR1, without experiencing any serious or unexpected side effects. Inhaled GSK1995057 conferred some protection against inflammation and endothelial injury from the subsequent challenge with a low dose of inhaled lipopolysaccharide (LPS). Significant reductions in the concentrations of inflammatory cytokines were also observed in bronchoalveolar lavage and serum [38]. In contrast, prophylactic treatment of a single dose of GSK2862277, a modified version of GSK1995057, did not reduce the postoperative alveolar capillary leak in patients following oesophagectomy [93]. The therapeutic potential of anti-TNFR1 dAb in ALI requires further investigation.

Antibody fragments with potential for inhalation therapy of asthma include VR942 (UCB4144, UCB Pharma, Brussels, Belgium) and CSJ117 (Novartis, Basel, Switzerland). VR942 is a dry powder formulation containing CDP7766, a mAb fragment targeting IL-13. Inhaled VR942 therapy leads to a rapid and durable inhibition of fractional exhaled nitric oxide (FeNO) and was generally well-tolerated in healthy and asthmatic subjects (NCT02473939) [45]. CSJ117 is a potent antagonist of human thymic stromal lymphopoietin (TSLP), an epithelial derived cytokine produced in response to proinflammatory stimuli [180]. In a Phase I study (NCT03138811), CSJ117 was administered as a PulmoSol™ engineered powder to adults with mild atopic asthma via DPI. Inhaled anti-TSLP was well-tolerated and attenuated bronchoconstriction post-allergen challenge [181]. A Phase II trial has been initiated in 2020 to investigate the efficacy and safety of CSJ117 for the treatment of severe uncontrolled asthma (NCT04410523). These data support further clinical development of inhaled antibody fragments as an alternative treatment to parenterally administered mAbs in asthma management.

Beyond mAb fragments, novel protein scaffolds such as Nanobodies^®^ are causing quite a stir of excitement in the field of protein-based therapeutics. Due to the smaller size and simpler structure of protein scaffolds, they have easier access to binding sites on enzymes, greater tissue penetration and accumulation, and enhanced thermostability in comparison to full-length mAbs [182]. In 2018, the first-in-class Nanobody^®^ caplacizumab (Cablivi^®^, Ablynx, Ghent, Belgium) received its first marketing authorisation from the European Medicines Agency for the treatment of acquired thrombotic thrombocytopaenic purpura [183]. While caplacizumab is for parenteral use, inhaled Nanobody^®^ platforms are currently under clinical development. The safety and tolerability of inhaled ALX-0171 was established in a first-in-infant Phase I/IIa clinical trials, and antiviral activity was observed [184]. Notwithstanding the completion of a Phase IIb dose-ranging study (NCT02979431), the development of ALX-0171 was abandoned, citing unsatisfactory evidence to support efficacy [185]. Due to the outbreak of COVID-19, a number of nanobodies are under development to combat SARS-CoV-2 infection and demonstrated potent neutralising activity in vitro, e.g., ty1 [108] and Nb11-59 [186]. Inhalable nanobodies candidates with desirable antiviral effect are anticipated to enter clinical study soon.

## 7. Future Prospects

The development and clinical use of biologics has expanded significantly in the past 15 years but options in terms of the route of administration for these drugs largely remain restricted to injection. Pulmonary delivery is an obvious non-invasive alternative for local and systemic delivery of biologics. Biologics have a complex structure and are prone to degradation under a variety of stresses encountered during the process of delivery via inhalation. Maintaining their structural integrity and biological activity is crucial for successful development of inhaled biologic formulations. The formulation design, excipient selection, processing technologies and inhalation devices should be systematically considered in product development Tissue penetration and systemic absorption of biologics are restricted by the high molecular mass and hydrophilic property. Understanding the transepithelial transport mechanism of macromolecules is essential for the development of inhaled biologics, particularly those intended for systemic therapy.

Moreover, structural modification of biomacromolecules to reduce molecular size, prolong the retention in the lung and improve receptor binding affinity are proven effective strategies that enhance the stability and permeability of inhaled biologics. Another major future focus should be on identifying biocompatible excipients to improve the stability, absorption and aerosol performance of inhaled biologics. Lessons learnt from Exubera^®^ clearly highlight that patient acceptance of the delivery system device is essential for successful commercial development of inhaled biologics, with an ideal inhalation device being small, portable, effective, convenient and easy to use. Overall, developing inhaled biologics is a systematic process and requires careful and holistic consideration of the disease, patient group(s), choice of therapeutic molecule and understanding its interaction with the biological barriers in the lung, as well as appropriate selection of formulation, excipients and devices.

## Figures and Tables

**Table 1:** Monoclonal antibodies approved by the FDA for the treatment of respiratory diseases.

Monoclonal Antibody	Brand Name	Target	Company	Approved Respiratory Indication(s)	Year of Initial Approval
Rituximab	Rituxan^®^	CD20	Genentech	GPA; MPA	1997
	Truxima^®^		Celltrion		2018
	Ruxience™		Pfizer		2019
Palivizumab	Synagis^®^	RSV envelope Fusion protein	AstraZeneca/AbbVie	Prophylaxis of RSV-associated serious lower respiratory tract disease	1998
Omalizumab	Xolair^®^	IgE	Genentech/Novartis	Moderate-to-severe persistent asthma	2003
Bevacizumab	Avastin^®^	VEGF	Genentech	Non-squamous NSCLC	2004
	Mvasi™		Amgen		2017
	Zirabev™		Pfizer		2019
Ipilimumab	Yervoy^®^	CTLA-4	BMS	NSCLC	2011
Raxibacumab	N.A.	*B. anthracis* PA	Emergent BioSolutions	Inhalational anthrax	2012
Nivolumab	Opdivo^®^	PD-1	BMS	NSCLC; SCLC	2014
Pembrolizumab	Keytruda^®^	PD-1	MSD	NSCLC; SCLC	2014
Ramucirumab	Cyramza^®^	VEGFR-2	Eli Lilly	Metastatic NSCLC	2014
Mepolizumab	Nucala^®^	IL-5	GSK	SEA, EGPA	2015
Necitumumab	Portrazza^®^	EGFR	Eli Lilly	Metastatic squamous NSCLC	2015
Atezolizumab	Tecentriq^®^	PD-L1	Genentech	NSCLC; SCLC	2016
Obiltoxaximab	Anthim^®^	*B. anthracis* PA	Elusys Therapeutics	Inhalational anthrax	2016
Reslizumab	Cinqair^®^	IL-5	Teva	SEA	2016
Benralizumab	Fasenra™	IL-5Rα	AstraZeneca	SEA	2017
Dupilumab	Dupixent^®^	IL-4Rα	Regeneron/Sanofi	Moderate-to-severe asthma, CRSwNP	2018
Durvalumab	Imfinzi^®^	PD-L1	AstraZeneca	NSCLC; SCLC	2017

Accessed from Drugs@FDA: FDA-Approved Drugs [site: https://www.accessdata.fda.gov/scripts/cder/daf] on 8 October 2020.CD: cluster of differentiation; CTLA-4: cytotoxic T-lymphocyte atigen-4; CRSwNP: chronic rhinosinusitis with nasal polyposis; EGFR: epidermal growth factor receptor; EGPA: eosinophilic granulomatosis with polyangiitis; FDA: U.S. Food and Drug Administration; GPA: granulomatosis with polyangiitis; IgE: immunoglobulin E; IL-4Rα: interleukin-4 receptor alpha; IL-5; interleukin-5; IL-5Rα: IL-5 receptor alpha; MPA: microscopic polyangiitis; NSCLC: non-small cell lung cancer; PA: protective antigen; PD-1: programmed cell death protein 1; PD-L1: programmed death ligand 1; RSV: respiratory syncytial virus; SCLC: small cell lung cancer; SEA: severe eosinophilic asthma; VEGF: vascular endothelial growth factor; VEGFR-2: VEGF receptor 2.

**Table 2:** Comparison of the key characteristics of biological and small-molecule drugs.

Characteristics	Biological Drugs	Small-Molecule Drugs
Molecular weight	>1 kDa, often much larger	Usually <700 Da
Molecular composition and structure	Proteins with known amino acid sequence, higher order structure, and diverse post-translational modifications	Organic chemicals with well-defined structure
Mechanisms of action	Fab-dependent binding to target epitope; Fc-dependent complement activation and effector cell recruitment	Typically enzyme or receptor inhibition
Pharmacological target	Normally extracellular	Extracellular or intracellular
Stability	Sensitive to physical conditions (e.g., temperature changes, shear stresses, pH, light) and proteases	Comparatively stable
Routes of administration	Mostly parenteral	Wide-ranging; oral route feasible if desired
In vivo half-life	Generally long; dosing intervals in weeks or months possible	Generally short; once-daily and more frequent dosing common
Specificity	High	Variable
In vivo degradation	Proteolytic metabolism	Enzymatic metabolism by cytochrome P450
Manufacturing process	Recombinant DNA technology; expansion in a unique cell line; purification from growth media	Chemical synthesis
Price	High	Variable
Me-too	Biosimilar (highly similar to a reference product)	Generic (identical entity)

**Table 3:** Selected preclinical studies on inhaled biological therapy for local and systemic disorders.

Drug	Target Disease	Formulation	Animal	Target/Format	Administration/Device	Reference & Year
Aldesleukin	Pulmonary metastases	Liposome	Dogs	IL-2	Puritan Bennet™ twin-jet nebuliser	[109,110] 1997
ALX-0171	RSV infection	Inhaled solution	Cotton rats	Anti-Fusion protein trivalent Nanobody^®^	AKITA^2^ APIXNEB^®^ nebuliser	[27,96] 2016
ALX-0171	RSV infection	Inhaled solution	New born lambs	Anti-Fusion protein trivalent Nanobody^®^	Aeroneb^®^ Solo system	[97] 2018
Anti-IL-17A PEG40-F(ab′)_2_ and Anti-IL-13 PEG40-Fab’	Asthma	Inhaled solution	NMRI mice	Anti-IL-17A F(ab’)_2_ and Anti-IL-13 Fab	Intranasal instillation	[111] 2014
Anti-IL-17A PEG40-Fab’	Asthma	Inhaled solution	Mice, rats and rabbits	Anti-IL-17A Fab	Intratracheal instillation	[37] 2017
Anti-IL-17A PEG20-Fab’, Anti-IL-17A PEG40-Fab’ and Anti-IL-13 PEG40-Fab’	Asthma	Inhaled solution	Mice	Anti-IL-17A and anti-IL-13 Fab	Intratracheal instillation	[60] 2018
Cetuximab	Lung tumour	Inhaled solution	Balb/c nude mice	Anti-EGFR mAb	Aeroneb Pro™ mesh nebuliser	[22,33] 2011
Cetuximab	Lung tumour	Inhaled solution	Balb/c nude mice and cynomolgus macaques	Anti-EGFR mAb	Microsprayer^®^ IA-1b aerosoliser	[21] 2014
CA154_582	Asthma	Inhaled solution	Balb/c mice	Anti-IL-13 Fab	inExpose nebulisation system	[88] 2012
CDP7766	Asthma	Inhaled solution	Cynomolgus macaques	Anti-IL-13 Fab	PARI eFlow^®^ mesh nebuliser	[89] 2017
EpoFc	Anemia	Inhaled solution	Cynomolgus monkeys	Erythropoietin Fc-fusion protein	Aeroneb Pro^®^ nebuliser	[57] 2004
FSHFc	Infertility	Inhaled solution	Cynomolgus monkeys	FSH Fc-fusion protein	Aeroneb Pro™ nebuliser	[81] 2005
GSK1995057	Acute lung injury	Inhaled solution	Cynomolgus monkeys	Anti-TNF receptor-1 dAb	Intratracheal instillation	[38] 2018
hGH	Growth hormone deficiency	SD powder	Wistar rats	hGH	Dry Powder Insufflator™	[65] 2004
Influenza subunit vaccine	Influenza	SD and SFD powder	Balb/c mice	Surface glycoprotein haemagglutinin	Dry Powder Insufflator™	[112] 2010
IFNβFc	Multiple sclerosis	Inhaled solution	Cynomolgus monkeys	IFNβ Fc-fusion protein	Aeroneb Pro^®^ mesh nebuliser	[82] 2012
IgG 43RCA-G1	Ricin intoxication	Inhaled solution	Balb/c mice/cynomolgus macaques	Anti-ricin mAb derived from scFv 43RCA	Micropipette tip and Aerogen^®^ Solo mesh nebuliser	[36] 2016
Infliximab	Asthma	SD powder	Balb/c mice	Anti-TNFα mAb	Dry Powder Insufflator™	[34] 2019
p55-specific dAb	Ventilator-induced lung injury	Inhaled solution	C57BL6 mice	Anti-p55 TNF receptor dAb	Intratracheal instillation	[91] 2012
PEG-rhα_1_-PI	Hereditary emphysema	Solution	CD1 mice	α1-PI	Intranasal instillation	[113] 2002
PEG12-IFNαPEG40-IFNα	Cancer or fibrosis	Inhaled solution	SD rats	PEGylated IFNα	Intratracheal instillation	[114] 2014

dAb: domain antibody; EGFR: epidermal growth factor receptor; FSH: follicle-stimulating hormone; hGH: human growth hormone, IFNβ: interferon beta; IL: interleukin; PEG: polyethylene glycol; RSV: respiratory syncytial virus; rhα_1_-PI: recombinant α_1_-proteinase inhibitor, scFv: single-chain variable fragment; SD: spray dried; SFD: spray freeze dried; TNF: tumour necrosis factor.

**Table 4:** Selected clinical studies and marketed products of inhaled biologic therapy.

Name	API	Development Stage	Company/Sponsor	Clinical Application	Delivery Device	Formulation	Reference, Year & Clinical Trial Number
**Inhaled proteins**							
Afrezza^®^	Insulin, 5.7 kDa	Marketed in 2014	MannKind	Diabetes mellitus	Dreamboat^®^ inhaler	Technosphere^®^ insulin inhalation powder	[187,188] 2014
Alpha-1 HC	Human α_1_-PI, 52 kDa	Phase II	Grifols Therapeutics	CF	AKITA^2^ APIXNEB^®^ nebuliser system	Inhaled solution	[158] 2016, NCT01684410
AZD1402/PRS-060	IL4 mutein (IL-4Rα antagonist), ~18 kDa	Phase I	AstraZeneca & Pieris Pharmaceuticals	Asthma	InnoSpire Go mesh nebuliser	Inhaled solution	[164,165] 2019, NCT03384290 and NCT03574805
Alteplase	rt-PA, 70 kDa	Phase II	University of Michigan & Genentech	Acute plastic Bronchitis	Nebuliser	Inhaled solution	[175] 2017, NCT02315898
ALX-009	OSCN-/bLF, 80 kDa	Phase I	Alaxia SAS	P. aeruginosa and Bcc infection in CF	Nebuliser	Inhaled solution	[174] 2018, NCT02598999
Alidornase alfa (PRX-110, AIR Dnase™)	rhDNase I, 37 kDa	Phase I	Protalix	CF	Philips Respironics I-neb AAD inhaler system	Inhaled solution	[155] 2017, NCT02605590
Dornase alfa (Pulmozyme^®^)	rhDNase I, 37 kDa	Marketed in 1993	Genentech	CF	Jet nebuliser/air compressor combinations	Inhaled solution	[189] 1996
Dornase alfa	rhDNase I, 37 kDa	Phase IV	Erasmus Medical Centre-Sophia Children’s Hospital	CF	AKITA^2^ APIXNEB^®^ nebuliser system	Inhaled solution	[128] 2011
Dornase alfa	rhDNase I, 37 kDa	Phase IV	PARI	CF	eRapid™ nebuliser system	Inhaled solution	[124] 2015, NCT01712334
DAS181 (Fludase^®^)	Recombinant sialidase fusion protein, 46 kDa	Phase I/II	Ansun BioPharma	Parainfluenza infection	Cyclohaler^®^ DPI	Dry powder	[154,167] 2015, NCT01037205, NCT01924793 NCT01113034
		Compassionate use/Phase III	Renmin Hospital of Wuhan University & Ansun BioPharma	COVID-19	Nebuliser	Inhaled solution	NCT04324489 NCT03808922
Exubera^®^	Insulin, 5.7 kDa	Marketed in 2006; withdrawn in 2007	Pfizer/Nektar Therapeutics	Diabetes mellitus	Exubera^®^ DPI inhaler	SD powder	[190] 2004
EpoFc	Epo Fc-fusion protein	Phase I	Syntonix Pharmaceuticals	Anemia	Aeroneb^®^ Pro nebuliser	Inhaled solution	[117] 2005
GM-CSF (Leukine^®^, Sargramostim)	rhuGM-CSF, 14 kDa	Phase I	Milton S. Hershey Medical Center	RASP	Nebuliser	Inhaled solution	NCT02601365
		Phase II	Children’s Hospital Medical Center, Cincinnati	PAP	Nebuliser	Inhaled solution	NCT01511068
		Phase II	Peking Union Medical College Hospital	PAP	Nebuliser	Inhaled solution	NCT02243228
Kamada AAT	Alpha-1-antitrypsin, 52 kDa	Phase II	Kamada	CF	PARI eFlow^®^ nebuliser	Inhaled solution	[160] 2009
Kamada-API	Alpha-1-antitrypsin, 52 kDa	Phase II/III	Kamada	AATD	PARI eFlow^®^ nebuliser	Inhaled solution	[161] 2017, NCT04204252
Pitrakinra (Aerovant^®^, AER-001)	IL4 mutein (IL-4Rα antagonist), 15 kDa	Phase II	Bayer	Asthma	PARI LC Plus nebuliser	Inhaled solution	[163] 2007, NCT00535031
Aldesleukin (Proleukin^®^)	Recombinant IL-2, 15 kDa	Phase I/II	M.D. Anderson Cancer Center	Lung metastases	Nebuliser	Inhaled solution	[173] 2000, NCT01590069
Survanta^®^	Bovine surfactant (phospholipids, triglycerides, free fatty acids and proteins, etc.)	Phase I/II	DMC Foundation	RDS	MiniHeart jet nebuliser	Inhaled solution	[191] 2019, NCT02294630
SNG001	Interferon-beta 1a, 22 kDa	Phase II	Synairgen	RVI in asthma	Nebuliser	Inhaled solution	[168,170] 2014, NCT01126177
		Phase II	Synairgen	COPD	Nebuliser	Inhaled solution	[169] 2019
		Phase II	Synairgen	COVID-19	Nebuliser	Inhaled solution	[171] 2020, NCT04385095
**Inhaled mAbs and mAb fragments**							
ALX-0171	Anti-F protein trivalent Nanobody^®^, 42 kDa	Phase II	Ablynx	RSV infection	FOX-Flamingo inhalation system	Inhaled solution	2017, NCT02979431 and NCT03418571
CSJ117	Anti-TSLP antibody fragment, 46 kDa	Phase I/II	Novartis	Asthma	Concept1 device (single dose DPI)	PulmoSol™ engineered powder	[181] 2020, NCT03138811 and NCT04410523
E25	Omalizumab, 149 kDa	Phase III	Genentech/Novartis	Asthma	PARI IS-2 nebuliser	Inhaled solution	[35] 1999
GSK1995057	Anti-TNFR1 dAb, 13 kDa	Phase I	GSK	Acute lung injury	PARI eFlow^®^ nebuliser	Inhaled solution	[38] 2018, NCT01587807
GSK2862277	Anti-TNFR1 dAb, 13 kDa	Phase II	GSK	Postoperative lung injury	PARI eFlow^®^ nebuliser	Inhaled solution	[93] 2020, NCT02221037
VR942/ CDP7766	Anti-IL-13 mAb fragment	Phase I	UCB Pharma	Asthma	Multidose F1P DPI	Dry powder	[45] 2018, NCT02473939

API: active pharmaceutical ingredient; AAT: alpha-1-antitrypsin; AATD: alpha-1-antitrypsin deficiency; bLF: bovine lactoferrin; Bcc: burkholderia cepacia complex; CF: cystic fibrosis; COPD: Chronic obstructive pulmonary disease; dAb: domain antibody; DPI: dry powder inhaler; Epo: erythropoietin; RDS: respiratory distress syndrome; RSV: respiratory syncytial virus; rhDNase I: recombinant human deoxyribonuclease I; IL-2: interlukin-2; OSCN-: Hypothiocyanite; PAP: pulmonary alveolar proteinosis; P. aeruginosa: pseudomonas aeruginosa; rt-PA: recombinant tissue plasminogen; rhuGM-CSF: recombinant human granulocyte-macrophage colony stimulating factor; RASP: respiratory virus-associated severe pneumonia; RVI: respiratory viral infection; SD: spray dried; TNFR1: tumour necrosis factor receptor-1; IL-4Rα: interleukin-4 receptor alpha; IL-13: interleukin-13; PI: protease inhibitor; TSLP: thymic stromal lymphopoietin.
